# Catalytic Efficiency of Chitinase-D on Insoluble Chitinous Substrates Was Improved by Fusing Auxiliary Domains

**DOI:** 10.1371/journal.pone.0116823

**Published:** 2015-01-23

**Authors:** Jogi Madhuprakash, Nour Eddine El Gueddari, Bruno M. Moerschbacher, Appa Rao Podile

**Affiliations:** 1 Department of Plant Sciences, School of Life Sciences, University of Hyderabad, Gachibowli, Hyderabad, India; 2 Department of Plant Biology and Biotechnology, Westphalian Wilhelm’s-University of Münster, Münster, Germany; George Washington University, UNITED STATES

## Abstract

Chitin is an abundant renewable polysaccharide, next only to cellulose. Chitinases are important for effective utilization of this biopolymer. Chitinase D from *Serratia proteamaculans* (*Sp*ChiD) is a single domain chitinase with both hydrolytic and transglycosylation (TG) activities. *Sp*ChiD had less of hydrolytic activity on insoluble polymeric chitin substrates due to the absence of auxiliary binding domains. We improved catalytic efficiency of *Sp*ChiD in degradation of insoluble chitin substrates by fusing with auxiliary domains like polycystic kidney disease (PKD) domain and chitin binding protein 21 (CBP21). Of the six different *Sp*ChiD fusion chimeras, two C-terminal fusions *viz.* ChiD+PKD and ChiD+CBP resulted in improved hydrolytic activity on α- and β-chitin, respectively. Time-course degradation of colloidal chitin also confirmed that these two C-terminal *Sp*ChiD fusion chimeras were more active than other chimeras. More TG products were produced for a longer duration by the fusion chimeras ChiD+PKD and PKD+ChiD+CBP.

## Introduction

Chitin is the second most abundant natural polysaccharide consisting of (1–4) linked units of 2-acetamido-2-deoxy-β-D-glucopyranose (GlcNAc) in a linear form. It is insoluble in water and primarily exists in two crystalline (*α*- and *β*-) forms. The *α*-chitin contains sheets of tightly packed alternating parallel and antiparallel chains [1] and is abundant in the exoskeletons of arthropods, insects, fungi and yeast cell walls. The chains are arranged in parallel orientation in *β*-chitin [2], which occurs less frequently in nature and is often extracted from squid pens. The insolubility of chitin is a major limitation for it to elicit response in biological systems. Chitooligosaccharides (CHOS) can be produced either by partial depolymerization of chitin, or by oligomerization of the basic monosaccharide building block *i*.*e*. GlcNAc. The available methods for depolymerization of chitin polymers can be classified into chemical, enzymatic [3], and other methods [4]. Chemical hydrolysis of chitin results in CHOS with low degree of polymerization (DP) generating high amount of GlcNAc [5]. Moreover, CHOS thus prepared are not ideal for bioactive studies due to the possible contamination with toxic chemicals. The chemical methods can be substituted by enzymatic methods for depolymerizing chitin and production of CHOS.

A great diversity of chitin degrading enzymes exists, including endo- and exo-acting chitinases. But, chitobiose is the major product of processive chitinases acting on chitinous substrates [6–7]. Thus, the generation of higher DP CHOS, suitable for biological applications, requires the use of a nonprocessive endo-chitinase of known specificity or a chitinase with transglycosylation (TG) activity. A few chitinases show TG activity along with hydrolytic activity [8–10], forming new glycosidic bonds between donor and acceptor saccharides. This property of chitinases can be used for the production of longer chain CHOS and also well-defined mixtures of CHOS with new or improved biological activity, by coupling smaller CHOS building blocks. The choice of starting substrate, enzyme and the processing time affect the outcome of enzymatic conversion of chitin/chitosan to CHOS [3, 11].

Chitinases (EC 3.2.1.14) belonging to glycosyl hydrolase family 18 (GH18) are the preferred biological tools for chitin degradation and production of soluble CHOS. To ensure efficient degradation of crystalline and inaccessible polymeric chitin substrates, chitinases often have one or more carbohydrate-binding modules (CBMs). The CBMs improve chitinase efficiency because they adhere to, and sometimes disrupt, the substrate [12]. The substrate binding properties of CBMs are known [13] but exactly how, and to what extent, CBMs contribute to the efficiency of substrate conversion is not completely clear. The CBMs may contribute to (i) correct positioning of the catalytic domain on to crystalline substrates, (ii) processive mode of action, and (iii) perhaps even local decrystallization of the substrate [14]. The presence of CBMs increases substrate affinity as well as efficiency of chitin hydrolysis, especially in hydrolysis of crystalline chitin forms [15–16]. In addition to CBMs, presence of other binding modules like polycystic kidney disease (PKD) domain is an added advantage for chitinase-mediated crystalline chitin hydrolysis. This domain has a β-sandwich fold, and the sequence “WDFGDG” is highly conserved. The role of PKD domain was analyzed for chitinase A from *Alteromonas* sp. strain O-7 (*Al*ChiA). Mutational studies proved that W30 and W67 in PKD domain of *Al*ChiA play an important role in efficient hydrolysis of powdered chitin [17].

Chitin-degrading organisms co-express few accessory proteins that disrupt the crystalline chitin substrates and increase the efficiency of chitinases. One such protein is the chitin binding protein 21 (CBP21), which showed a strong affinity to β-chitin [18] and catalyzed cleavage of glycosidic bonds in the crystalline β-chitin nanowhiskers [19]. CBP21 was a member of CBM family-33 (CBM33) and the best example of lytic polysaccharide monooxygenases (LPMOs). Proteins with CBM33 were recently grouped under family “Auxiliary Activity-10” (AA10) [20]. Similar enzymatic activity was demonstrated for other members of the CBM33 family including those active on cellulose [21].

**Figure 1:**
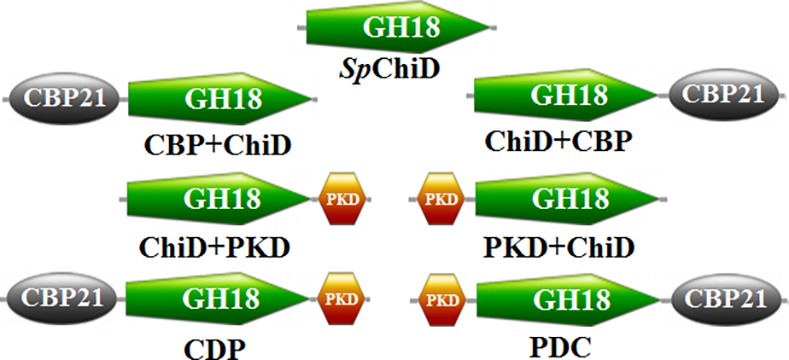
Schematic representation of domain organization. *Sp*ChiD fusion chimeras generated using overlap extension PCR. The designation of chimeras was based on the type of domain fused and its orientation.

Chitinase D from *Serratia proteamaculans* (*Sp*ChiD) showed unique combination of hydrolytic and hyper TG activities [9]. *Sp*ChiD binds to polymeric substrates with feeble hydrolytic activity as it contained only the catalytic GH18 domain and no auxiliary domains. We made an attempt to increase the catalytic efficiency of *Sp*ChiD, for degradation of insoluble chitin substrates, by fusing the auxiliary domains like PKD and CBP21 to the ‘N’ or ‘C’ or ‘N’ and ‘C’ termini. Domain organization of different *Sp*ChiD fusion chimeras generated for this study can be seen in Fig. 1. The chimeras appended with auxiliary domains at their C-terminus showed enhanced hydrolytic activity on insoluble polymeric chitin substrates. Fusion of auxiliary domains also improved the TG activity of the chimeras ChiD+PKD and PKD+ChiD+CBP (PDC) with DP4 substrate.

## Materials and Methods

### Generation of *Sp*ChiD fusion chimeras

Overlap extension/fusion PCR was performed to generate *Sp*ChiD fusion chimeras as described by Neeraja et al. [22]. Plasmid templates pChiD-pET22b(+), pChiA-pET22b(+) and pCBP21-pET22b(+) with appropriate combinations of primers (S1 Table) were used to generate *Sp*ChiD fusion chimeras. Based on the fusion of auxiliary domains to either ‘N’ or ‘C’ or ‘N’ and ‘C’ termini of the *Sp*ChiD gene, the chimeras were designated as CBP+ChiD, ChiD+CBP, PKD+ChiD, ChiD+PKD, PKD+ChiD+CBP (PDC), and CBP+ChiD+PKD (CDP) (Fig. 1). The amplicons (CBP+ChiD & ChiD+CBP of 1.7 kb, PKD+ChiD & ChiD+PKD of 1.6 kb, PDC & CDP of 2.1 kb) were double-digested and ligated to *Nco* I & *Xho* I sites of pET-22b (+) expression vector. All the ligation reactions were performed at 16°C for 16 h, using T4 DNA ligase. Highly efficient competent cells of *Escherichia coli* Rosetta-gami II (DE3) were used for transformation. Positive clones were selected on appropriate antibiotic plates and confirmed by both double digestion and sequencing.

### Protein expression, isolation and purification

#### Heterologous expression

*E*. *coli* Rosetta-gami II (DE3) cells containing appropriate *Sp*ChiD fusion chimeras were used for over expression of protein. Colonies harbouring desired plasmids were inoculated into the LB medium with ampicillin and chloramphenicol at 100 μg/mL and 25 μg/mL, respectively. Cultures were grown overnight at 37°C and 200 rpm. One percent overnight culture was used for over expression of proteins. The cultures were grown to an optical density of 0.4–0.6 at 600 nm (OD_600_), and IPTG was added to a final concentration of 0.5 mM. The cells were further incubated for 16 h at 18°C, followed by harvesting of cultures by centrifugation at 7741×g for 10 min at 4°C.

#### Protein isolation

Periplasmic fraction (PF) was prepared as suggested in the pET expression system manual, Novagen, with little modifications. Cells were subjected to an osmotic shock in a two-step procedure for isolating PF. In the first step, the cell pellet was resuspended in 15 ml of ice-cold spheroplast buffer and incubated at 4°C with gentle mixing for 15 min. The PF buffer-1 contains 10 ml of 1 M Tris–HCl, pH 8.0, 20 g sucrose, 200 μL 0.25 M EDTA, pH 8.0, and 200 μL 50 mM phenylmethylsulfonyl fluoride (PMSF) with a final volume adjusted to 100 mL using distilled water. The cells were harvested by centrifugation at 7741×g, for 8 min at 4°C, and the supernatant was discarded. The pellet was further resuspended in 15 mL of ice-cold filter- sterilized PF buffer-2 containing 5 mM MgSO_4_ and incubated at 4°C for 10 min, followed by centrifugation at 7741×g, for 8 min at 4°C. The supernatant was sterilized using 0.2 μm filters and used for purification.

#### Ni-NTA purification

Before purification, the protein in the PF was buffer exchanged against the lysis buffer (50 mM NaH_2_PO_4_, 300 mM NaCl, 10 mM imidazole with pH 8.0), which was used as equilibration buffer in further steps of affinity purification. Six mL ethanol suspension of the Ni-NTA agarose was packed into a sterile 10 mL syringe. Column was equilibrated with lysis buffer, followed by PF application onto the column. Flow through was collected and the column was washed with four column volumes of wash buffer (50 mM NaH_2_PO_4_, 300 mM NaCl, 20 mM imidazole with pH 8.0). Bound recombinant protein was eluted in a gradient method described by Purushotham et al. [23] and purity of the protein fractions was assessed by performing 12% SDS-PAGE.

### Preparation of the protein and zymogram analysis

Protein fractions with highest purity were pooled and concentrated using Macrosep Centrifugal Devices (Pall Corporation, USA) with 10 kDa cut-off. Concentrated protein was dialysed against 50 mM sodium phosphate pH 8.0. The purified proteins were quantified using Pierce BCA protein assay kit (Thermo Scientific, USA). Slope from the standard calibration curve of bovine serum albumin (BSA) was used for quantification of protein. Dot blot assay was performed to detect the activity of purified *Sp*ChiD fusion chimeras as described by Purushotham et al. [23]. The lytic zones were visualized as dark blue spots in the gels under UV transilluminator.

### Reducing end assay

Chitinase activity was measured by reducing-end assay for the quantification of CHOS as described by Neeraja et al. [22] with slight modifications. All the reactions were performed in triplicate with 200 μL reaction volume. Appropriate concentration of *Sp*ChiD fusion chimeras was incubated with colloidal chitin in 50 mM sodium phosphate pH 8.0 at 40°C, 200 rpm for 1 h. Incubation was followed by centrifugation of the reaction mixture at 13,600×g at 4°C for 15 min. A 40 μL of the clear supernatant containing reducing sugars was mixed with 300 μL of the freshly prepared color reagent (0.5 M sodium carbonate, 0.05% potassium ferricyanide) and boiled for 20 min at 100°C in dark. Two hundred microliters from each reaction was taken in to a 96 well microtiter plate and the OD_420_ was measured using microtiter plate reader (Multiscan, Labsystems, Finland). One unit was defined as the amount of enzyme that liberated 1 μmol of reducing sugar per second.

### Steady-state kinetics

Kinetic parameters for all the *Sp*ChiD fusion chimeras were obtained by incubating different concentrations of colloidal chitin (0–50 mg/mL) as substrate, with 38 μg of enzyme in 50 mM sodium phosphate, pH 8.0. Proper controls for buffer, enzyme and substrate alone were maintained. The reactions were carried out at 40°C for 1 h, with constant shaking at 200 rpm. Enzyme activity was measured as described above, and the unit was defined as the release of 1 μmol of GlcNAc per second under standard experimental conditions. Specific activity was calculated in nkat/mg of protein. Kinetic values were obtained from three independent sets of data fitting to the Michaelis-Menten equation by nonlinear regression function available in GraphPad Prism software version 5.0 (GraphPad Software Inc., San Diego, CA).

### Time course of colloidal chitin hydrolysis

Time-dependent degradation of colloidal chitin by native *Sp*ChiD and its fusion chimeras was performed by incubating 25 mg/mL of colloidal chitin with 38 μg of the respective enzyme in 50 mM sodium phosphate, pH 8.0. To check the synergistic activity of CBP21, an additional control reaction with *Sp*ChiD and CBP21 was set up and referred to as synergy. Reaction mixtures were incubated at 40°C and 800 rpm in a thermomixer. A 100 μL of the reaction mixture was withdrawn at regular intervals (30, 60, 90, 120, 150, 180, 210, 360 and 720 min) into sterile 1.5 mL eppendorf tubes and centrifuged at 16,100×g to get a clear supernatant containing soluble CHOS. Enzyme activity was measured as described above. All assays were performed in triplicate with proper blanks (buffer + 25 mg/mL colloidal chitin) and controls (buffer alone and buffer + 38 μg of enzyme). Micromoles of the reducing ends released were calculated using the slope from the standard graph plotted with different concentrations of GlcNAc.

### Degradation of ‘α’ and ‘β’ chitin

The polymeric substrates α-chitin (from shrimp shells) and β-chitin (from squid pen) were kindly provided by Dr. Dominique Gillete, Mahtani Chitosan, Veraval, India. The substrate α-chitin (or) β-chitin (2.5%, w/v) was incubated with 1 μM each of *Sp*ChiD or its fusion chimeras, at 40°C for 1 h, in 50 mM sodium acetate buffer, pH 5.2. After incubation, the reaction mixtures were spun down at 4,200×g and the clear supernatant was used for reducing end assay. One unit was defined as the amount of chitinase that liberated 1 μmol of reducing sugar per second.

### High performance liquid chromatography (HPLC)

*Sp*ChiD fusion chimeras, positive in zymogram analysis, were considered for HPLC analysis, to assess the effect of domain fusions on the hydrolytic and TG activities of *Sp*ChiD. One mM chitotetraose (DP4) was incubated with 350 nM of the purified *Sp*ChiD or its fusion chimeras. Reaction was performed in 20 mM sodium phosphate buffer, pH 8.0, at 40°C. Fractions were collected at regular intervals and the reaction was stopped using equal volume of 70% acetonitrile. Reaction mixture (20 μL) from each fraction was injected into HPLC (Shimadzu, Tokyo, Japan) using Hamilton (Hamilton Bonaduz, Switzerland) syringe. The products were analysed on SHODEX Amino-P50 4E column (4.6 ID x 250 mm, Showa Denko K.K, USA) through isocratic elution using 70% acetonitrile, with a flow rate of 0.7 mL/min and the eluted CHOS were monitored at 210 nm. CHOS with DP1-DP6 were quantified as described by Madhuprakash et al. [24].

### Statistical analyses

The reported values are based on mean ±SD of three identical experiments. Data were analyzed by one-way or two-way ANOVA using GraphPad Prism software version 5.0 (GraphPad Software Inc., San Diego, CA). The multiple mean comparisons were performed using Tukey's Multiple Comparison Test or Bonferroni post-tests. Statistical significance was determined at p≤0.05.

## Results and Discussion

### Expression, purification and dot blot assay

**Figure 2:**
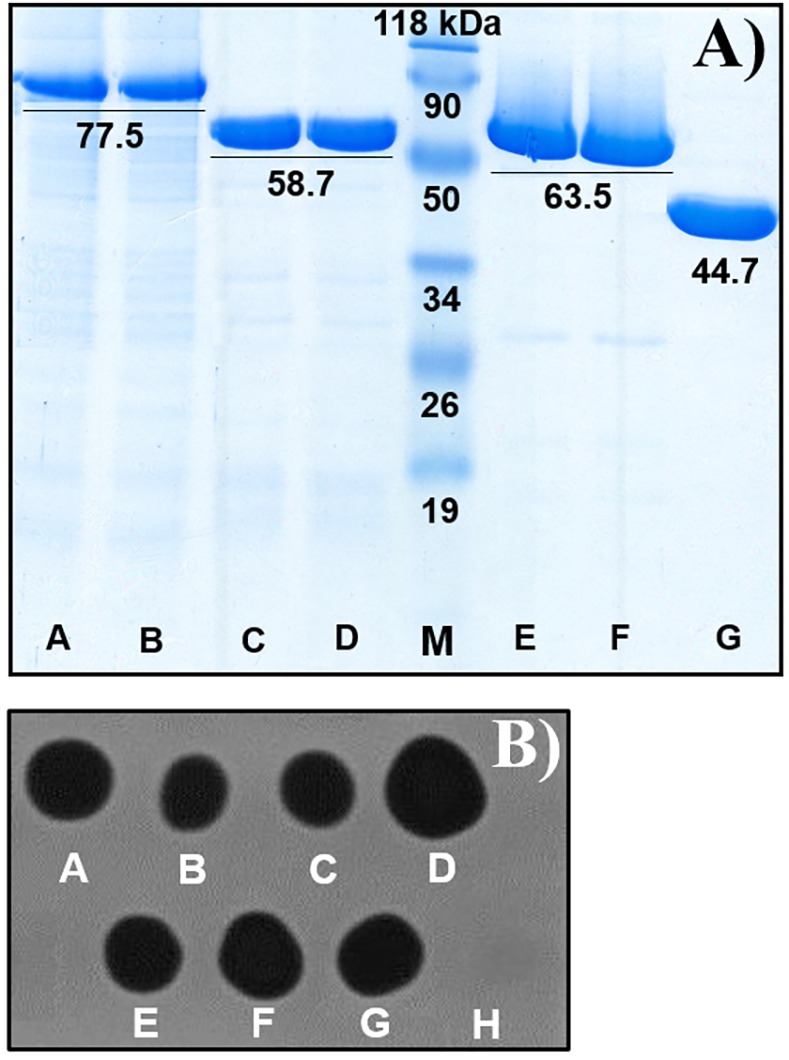
Purification and activity analysis of *Sp*ChiD fusion chimeras. (A) Recombinant *Sp*ChiD and its fusion chimeras were purified by Ni-NTA affinity chromatography and loaded on 12% SDS-PAGE, followed by staining with Coomassie brilliant blue G-250. The molecular weight of all the proteins and the standards was indicated in kDa, below or above the corresponding band. M: Pre-stained protein molecular weight marker. Lanes A-G: Purified proteins PDC, CDP, PKD+ChiD, ChiD+PKD, CBP+ChiD, ChiD+CBP and *Sp*ChiD, respectively. (B) Purified *Sp*ChiD and its fusion chimeras were spotted (5 μg) on glycol chitin substrate containing polyacrylamide gel and incubated overnight at 37°C in humid chamber. After incubation, the gel was stained with 0.01% Calcofluor white M2R for 10 min at 4°C. The gel was placed on UV transilluminator to visualize lytic zone. Spots A-F were due to the activity of fusion enzymes PDC, CDP, PKD+ChiD, ChiD+PKD, CBP+ChiD and ChiD+CBP, respectively. (G) *Sp*ChiD as positive control; (H) buffer as negative control.

*E*. *coli* Rosetta-gami II (DE3) cells harbouring the plasmids of *Sp*ChiD and the corresponding fusion chimeras were used for protein expression. PF was isolated from the harvested cell pellets and the soluble proteins of *Sp*ChiD and its fusion chimeras in the PFs were purified using Ni-NTA affinity chromatography. SDS-PAGE analysis confirmed the purity of collected protein fractions that matched the expected molecular mass of *Sp*ChiD (~ 44.7 kDa) and its chimeras CBP+ChiD & ChiD+CBP (~ 63.5 kDa), PKD+ChiD & ChiD+PKD (~ 58.7 kDa), PDC & CDP (~ 77.5 kDa) (Fig. 2A). The dark blue spots in the in gel assay confirmed the activity of *Sp*ChiD and its fusions (Fig. 2B).

### Kinetic analysis

**Figure 3:**
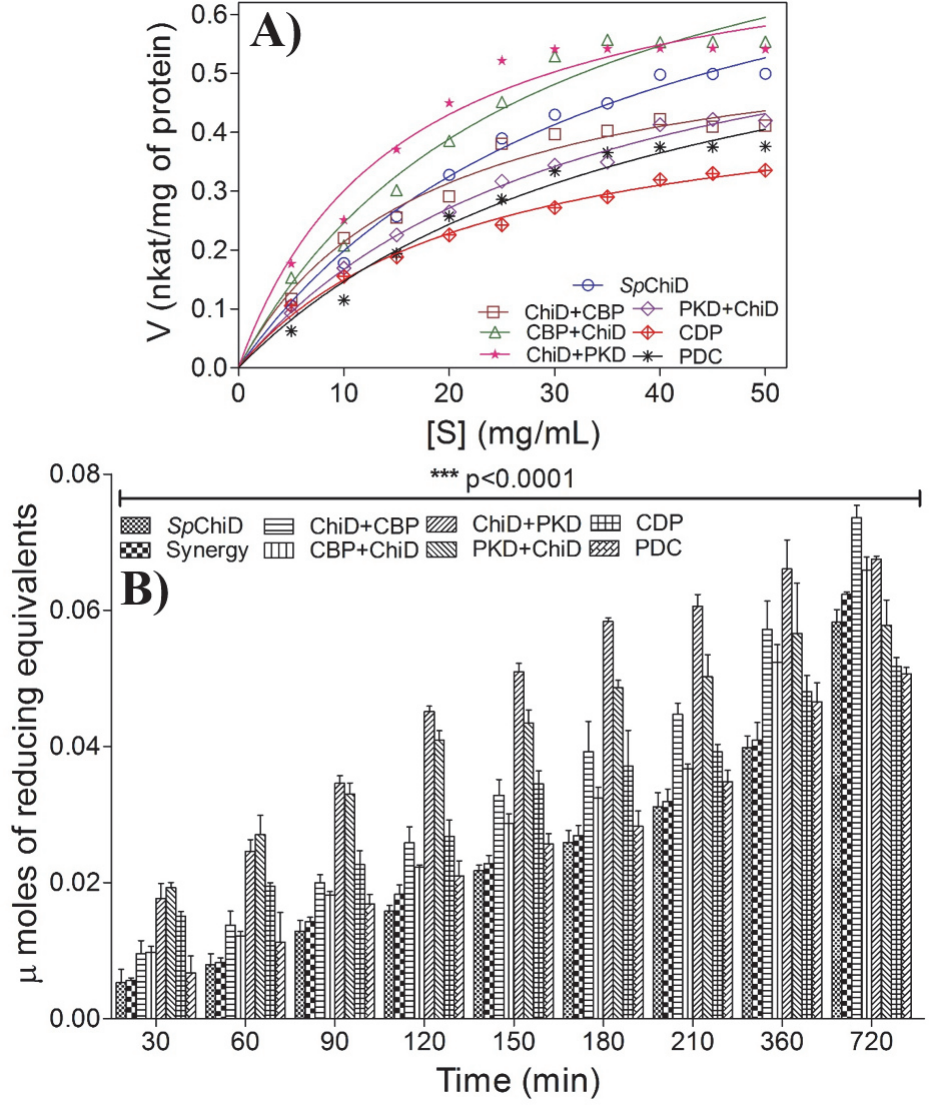
Characterization of *Sp*ChiD fusion chimeras. (A) Different concentrations of colloidal chitin (0–50 mg/mL) were incubated with *Sp*ChiD and its chimeras in 50 mM sodium phosphate buffer pH 8.0, with respective controls in triplicate at 40°C for 1 h at 200 rpm. Specific activity in nkat/mg of protein was calculated and plotted against substrate concentration. The data was fitted to the Michaelis–Menten equation by nonlinear regression function using GraphPad Prism software version 5.0, to obtain the respective kinetic graphs and values. (B) Time-dependent degradation of colloidal chitin by native *Sp*ChiD and its fusion chimeras was performed by incubating 25 mg/mL of colloidal chitin with 38 μg of each fusion enzyme in 50 mM sodium phosphate, pH 8.0. Reaction mixtures were incubated at 40°C, in a thermomixer at 800 rpm. The values are based on mean ±SD of three identical experiments. Data were analyzed by two-way ANOVA followed by Bonferroni post-tests. Statistical significance was determined at p≤0.05.

The kinetic parameters for the enzyme *Sp*ChiD and its fusion chimeras were determined using colloidal chitin as the substrate (Fig. 3A). The derived kinetic values (*K*_m_, *V*_max_, *K*_cat_ and *K*_cat_/*K*_m_) of the fusion chimeras were compared against *Sp*ChiD and presented in Table 1. Among the six *Sp*ChiD fusion chimeras, two C-terminal fusions ChiD+CBP, ChiD+PKD and one N-terminal fusion CBP+ChiD showed decreased *K*_m_ of 17.6, 15.2 and 27.4 mg/mL, respectively, when compared to *Sp*ChiD (35.1 mg/mL), indicating increased affinity towards colloidal chitin. Higher ligand-binding capacity/affinity did not improve cellulase activity, as the ultra-tight binding could become a restriction for the dynamic motion of the enzymes [13]. In line with this observation, though the fusion chimera CDP had a lower *K*_m_ of 22.4 mg/mL, the overall catalytic efficiency was low (24.86 s^-1^ mg^-1^ mL) compared to *Sp*ChiD (29.33 s^-1^ mg^-1^ mL). The order of overall catalytic efficiency (s^-1^ mg^-1^ mL) of *Sp*ChiD and its fusion chimeras was as follows: ChiD+PKD (57.4) > ChiD+CBP (38.5) = CBP+ChiD (38.6) > *Sp*ChiD (29.3) > PKD+ChiD (25.1) ≈ CDP (24.8) > PDC (21.1).

**Table 1:** Kinetic parameters of *Sp*ChiD fusion chimeras.

Enzyme	*K*_m_ (mg/mL)	*V*_max_ (nkat/mg of ptn)	*K*_cat_ (s^-1^)	*K*_cat_/*K* (s^-1^ mg^-1^ mL)
*Sp*ChiD	35.12	0.8964	10.3×10^2^	29.33
ChiD+CBP	17.60	0.5903	6.80×10^2^	38.54
CBP+ChiD	27.40	0.9213	10.6×10^2^	38.64
ChiD+PKD	15.15	0.7564	8.70×10^2^	57.37
PKD+ChiD	32.67	0.7141	8.20×10^2^	25.12
CDP	22.37	0.4838	5.60×10^2^	24.86
PDC	39.48	0.7248	8.30×10^2^	21.09

GHs degrade crystalline polysaccharides in a relatively less efficient manner as their target glycosidic bonds are often inaccessible to the active site. To overcome these problems, many of the GHs that hydrolyse insoluble substrates are often multi-modular, comprising catalytic modules appended to one or more auxiliary domains like CBMs, PKD and fibronectin III (FnIII). These auxiliary domains increase the binding affinity towards soluble and insoluble carbohydrate substrates. Single-module polysaccharide hydrolases such as cellobiohydrolase [25] and mannanase [26] exhibit higher catalytic activity as well as thermostability when attached to heterologous CBMs. These auxiliary domains were thought to bring the catalytic domains in close proximity to the substrates and in turn improve the overall catalytic efficiency of enzymes. We showed for the first time enhancement of hydrolytic activity on insoluble chitin polymer, for a single module GH18 chitinase, with fusion of PKD or CBP21.

### Time course of colloidal chitin hydrolysis

The efficiency of *Sp*ChiD fusion chimeras in degrading insoluble polymeric substrate like colloidal chitin was tested in a time course study. Equimolar concentration of fusion chimeras was used for degradation of colloidal chitin (25 mg/mL) and the activities were compared with *Sp*ChiD. CBP21 was known to act synergistically with the chitinases enhancing the efficiency of crystalline chitin hydrolysis [18, 27]. In the present study, as the *Sp*ChiD fusion chimeras were prepared in combination with CBP21, a positive control was maintained to check the synergism, between CBP21 and *Sp*ChiD (Synergy). The chimeras fused with CBP21 alone were more active compared to *Sp*ChiD (Fig. 3B). The addition of CBP21 had only minor effect on the activity of *Sp*ChiD in colloidal chitin degradation, compared to the fusion chimeras generated with CBP21 alone. Minor differences were observed between the fusions, ChiD+CBP and CBP+ChiD in the initial rates of degradation up to 90 min. From 120 min, the difference in the activity of these chimeras was high, with the C-terminal fusion (*i*.*e*. ChiD+CBP) showing more activity (Fig. 3B). After 720 min of incubation, the chimeras fused with CBP21 alone had higher activity compared to *Sp*ChiD and synergy (*Sp*ChiD and CBP21).

Chimeras with PKD alone and CDP were highly active from 30 min of incubation, when compared to chimeras with CBP21 alone. The difference between the activity of ChiD+PKD and PKD+ChiD was less up to 90 min, in spite of high initial rates of degradation, which was similar to the CBP21 alone fusion chimeras (Fig. 3B). But, at the end of 720 min, out of the six fusion chimeras analyzed, ChiD+CBP was more active. The fusion chimera, CDP appeared to be more active than PDC up to 210 min. At later time points, 360 and 720 min, there was no major difference in the activity of CDP and PDC (Fig. 3B). Time-dependent degradation studies with colloidal chitin also revealed that the C-terminal *Sp*ChiD fusion chimeras *i*.*e*. ChiD+CBP and ChiD+PKD were more active than other chimeras.

### Hydrolysis of ‘α’ or ‘β’ chitin by *Sp*ChiD fusion chimeras

**Figure 4:**
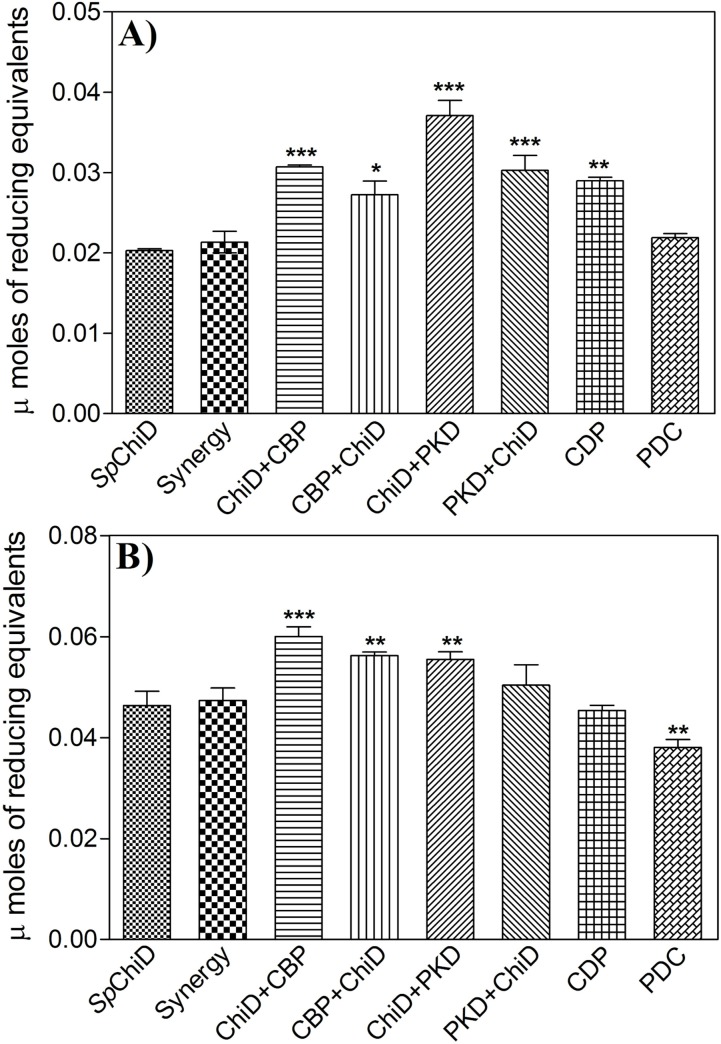
Degradation of α- or β-chitin by *Sp*ChiD fusion chimeras. (A) α-chitin (or) (B) β-chitin (2.5% (w/v)) was incubated with 1μM each of *Sp*ChiD or its fusion chimeras at 40°C for 1 h, in 50 mM sodium acetate buffer, pH 5.2. The values are based on mean ±SD of three identical experiments. Data were analyzed by one-way ANOVA followed by Tukey's Multiple Comparison Test. Statistical significance was determined at p≤0.05.

The effect of fusion of binding domains (CBP or PKD) with *Sp*ChiD on α- or β-chitin degradation was also analyzed. All the chimeric enzymes were highly active on β-chitin compared to α-chitin, under standard reaction conditions. Among all the fusion chimeras, the two C-terminal fusions, *i*.*e*. ChiD+PKD and ChiD+CBP were more active on α- and β-chitin (Fig. 4A and B), respectively. This was consistent with the substrate preferences of the appended auxiliary domains [17–18]. The order of hydrolytic activity on α-chitin was as follows: ChiD+PKD > ChiD+CBP = PKD+ChiD > CDP ≈ CBP+ChiD > PDC ≈ Synergy ≈ *Sp*ChiD. But, on β-chitin the activity of chimeric enzymes had a different order *i*.*e*. ChiD+CBP > CBP+ChiD ≈ ChiD+PKD > PKD+ChiD > Synergy ≈ *Sp*ChiD ≈ CDP > PDC. The addition of CBP21 in reaction had only a minor effect on the *Sp*ChiD-mediated degradation of colloidal/α-/β-chitin, compared to the CBP21 fused chimeras. Enhanced hydrolytic activity of CBP21 fused chimeras on insoluble chitin polymers can be explained in terms of intramolecular synergism. This mechanism was initially reported for endoglucanase A (CenA) from *Cellulomonas fimi* that had a catalytic domain and a non-hydrolytic cellulose-binding domain which can function independently. The individual domains interact synergistically in the disruption and hydrolysis of cellulose fibers [28]. CBP21 with its enzymatic activity on β-chitin nanowhiskers was also functionally independent [19]. Thus, the addition of CBP21 for improving catalytic efficiency of chitinases can be considered as an example for intermolecular synergism, whereas, the CBP21 fusion chimeras as examples for intramolecular synergism. There could be a competition for the binding sites in substrate between the two individual protein molecules (intermolecular synergism), which may decrease the overall catalytic efficiency [28].

Hydrolysis of polymeric substrates like colloidal/α-/β-chitin with low efficiency by *Sp*ChiD may not be entirely due to the differences in the binding or absence of auxiliary binding domains. The interactions with aromatic residues located at the catalytic domains of chitinases probably are essential for substrate binding by *Sp*ChiD, because, Trp69, Trp33, and Trp245 of *Sm*ChiA were crucial for optimum binding [29]. Of these three residues, Trp69 and Trp33 were located in the N-terminal PKD domain, whereas, Trp245 was located in the catalytic domain, but, all three residues were proposed to act cooperatively in the chitin binding by *Sm*ChiA [29]. Thus, *Sm*ChiA and *Al*ChiA were active on crystalline α-chitin compared to other chitinases produced by the respective bacterial species [17, 30]. The increased activity of ChiD+PKD fusion chimera may be due to the arrangement of aromatic residues on the surface of the chimeric protein, both from the PKD domain and the catalytic domain of *Sp*ChiD. Such a linear arrangement of aromatic residues is crucial for carbohydrate degrading enzymes, to tether to the crystalline polysaccharides, guiding sugar chains towards the catalytic center and to display increased catalytic efficiencies (Uchiyama et al., 2001).

### Effect of fusions on TG activity of *Sp*ChiD with DP4 substrate

#### ChiD+CBP and CBP+ChiD

**Figure 5:**
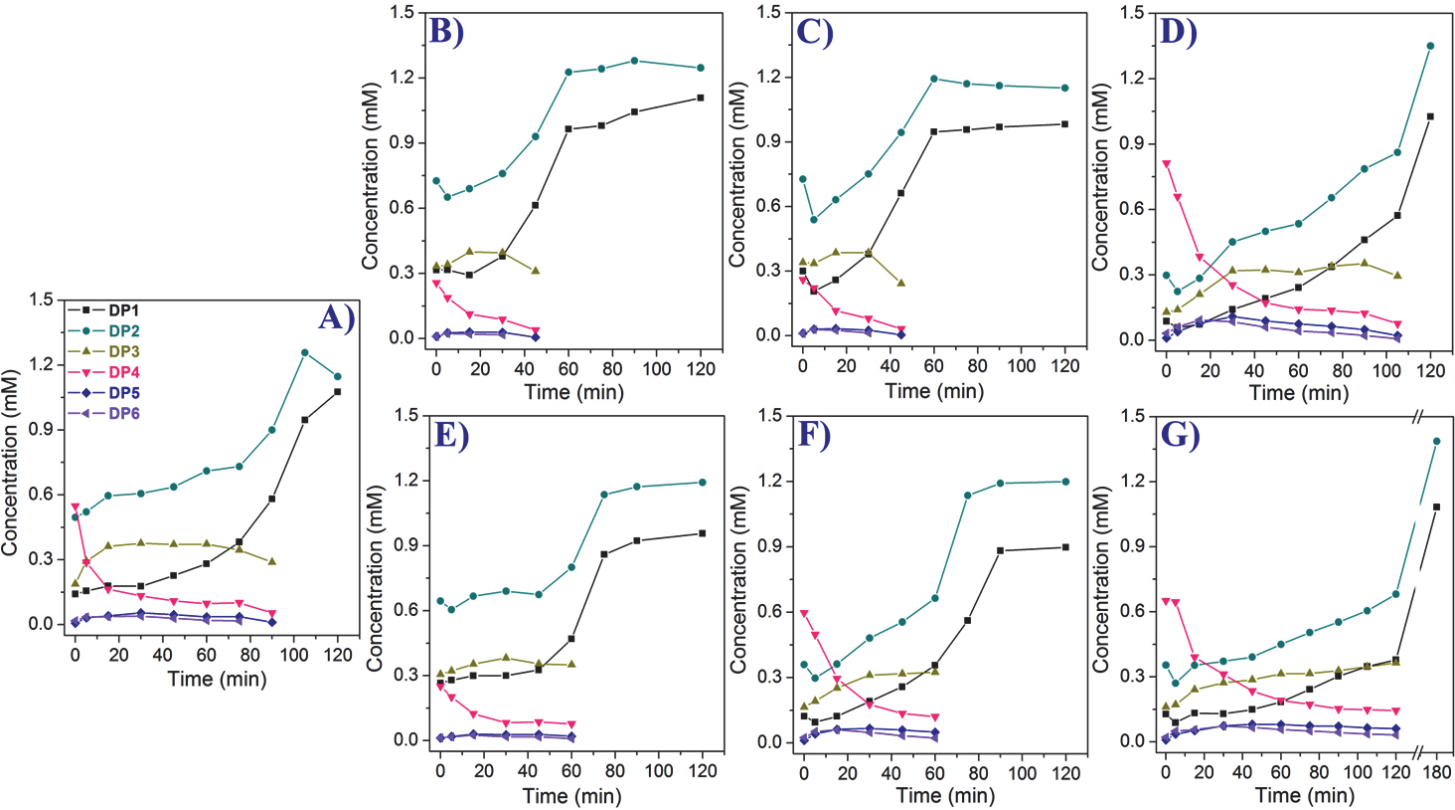
Product profiles of the *Sp*ChiD fusion chimeras. HPLC quantification profiles for the *Sp*ChiD fusion chimeras represent the hydrolytic (DP1-DP3) and quantifiable TG (DP5, DP6) products accumulated during the course of reaction with 1 mM DP4 substrate. All the reactions were performed at 40°C, in 50 mM sodium phosphate buffer, pH 8.0. (A) *Sp*ChiD, (B) ChiD+CBP, (C) CBP+ChiD, (D) ChiD+PKD, (E) PKD+ChiD, (F) CDP and (G) PDC.

The fusion chimeras ChiD+CBP and CBP+ChiD showed more of hydrolysis than TG activity compared to *Sp*ChiD (Fig. 5A-C). A rapid decrease in the initial DP4 substrate was observed and from 60 min only DP1 and DP2 were detected (Fig. 5B and C). Quantifiable TG products, DP5 and DP6, were detected in very low quantity at all the time points, except at 5 min where, a minor increase in the TG products was observed. ChiD+CBP and CBP+ChiD produced DP5 in proportions 1.7 and 2.3%, whereas, DP6 in proportions 1.5 and 2.1%, respectively, at 5 min. After 5 min, the TG products decreased with time and only DP5 was detected at the end of 45 min for both the chimeras. The fusion of only CBP21 either at N- or C-terminus did not result in increase of TG activity, rather decreased when compared to *Sp*ChiD.

#### ChiD+PKD and PKD+ChiD

The C-terminal PKD fusion chimera *i*.*e*. ChiD+PKD showed an improved TG, in terms of quantity of TG products produced and also in the extended duration of TG, up to 105 min (Fig. 5D). TG products were detectable from 0^th^ min, with more of DP6 (2.4%) than DP5 (0.8%). At 15 min, the quantity of DP5 and DP6 products generated by ChiD+PKD increased to 6.9 and 8.3%, respectively. But, these relative proportions of TG products was reversed by the end of 30 min, with DP5 and DP6 products detected in quantities 8 and 6.3%, respectively. The quantity of TG products with ChiD+PKD was 2-fold high compared to *Sp*ChiD, which produced maximum TG products at 30 min (DP5–3.9% and DP6–2.8%). The fusion chimera PKD+ChiD had a product profile similar to ChiD+CBP or CBP+ChiD, with more of hydrolysis than TG (Fig. 5E). The chimera, PKD+ChiD showed maximum TG activity at 15 min with DP5 and DP6 products in proportions 2 and 1.7%, respectively. Later, the TG products decreased and were detectable up to 60 min only. At the end of 75 min, only DP1 (42%) and DP2 (58%) products were detectable.

#### CDP and PDC

Both CDP and PDC produced more TG products compared to *Sp*ChiD. At 15 min, CDP produced equal quantity of DP5 and DP6 products, which constituted 5.4 and 5.2% of the products, respectively. The concentration of DP5 remained unaltered till the end of 30 min, but, a decrease in the quantity of DP6 (3.8%) was observed. TG products were detectable up to 60 min only for CDP with DP5 and DP6 in proportions 3.2 and 1.5%, respectively (Fig. 5F). Similar to CDP, PDC also produced equal quantity of DP5 (4.2%) and DP6 (4.6%) products at 15 min and these proportions increased to 6 and 5.8%, respectively by the end of 30 min. There was a decrease in the quantity of TG products from 90 min. The TG activity displayed by PDC at 120 min (Fig. 5G) was equivalent to the optimum TG activity of *Sp*ChiD at 30 min.

**Figure 6:**
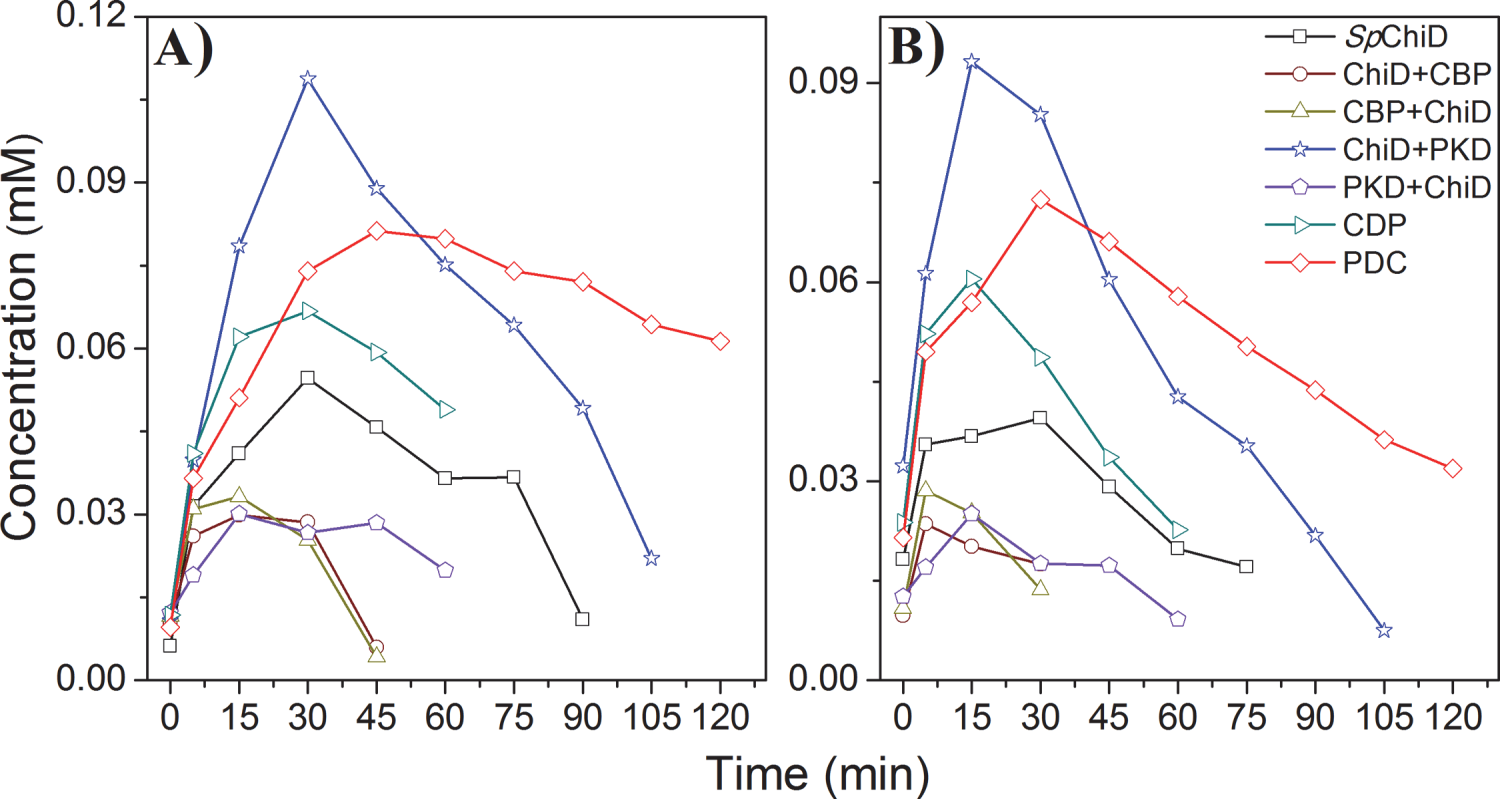
Comparison of quantifiable TG products produced by *Sp*ChiD and its fusion chimeras. TG products DP5 (A) and DP6 (B) were generated by *Sp*ChiD or its six fusion chimeras with 1 mM DP4 substrate in 20 mM sodium acetate buffer pH-5.6 at 40°C. Product quantification was done by a linear correlation between peak area and concentration of chitooligosaccharides in standard samples.

The fusion chimeras ChiD+CBP, CBP+ChiD and PKD+ChiD produced very low quantity of TG products (DP5 and DP6) compared to *Sp*ChiD, except the C-terminal PKD fusion *i*.*e*. ChiD+PKD, which had an improved TG (Fig. 6). The dual-domain fusions (*i*.*e*. CDP & PDC), which had low catalytic efficiency towards insoluble polysaccharides, produced higher amounts of TG products with DP4 substrate compared to *Sp*ChiD. But, PDC displayed efficient TG than CDP and *Sp*ChiD both in terms of quantity of TG products produced and duration of TG activity. The reasons behind increase or decrease in the TG activity of *Sp*ChiD fusion chimeras with soluble CHOS like DP4 need to be analyzed.

## Conclusion

ChiD+PKD had an improved hydrolytic activity on α-chitin, whereas, ChiD+CBP was more active on β-chitin. Time-course degradation with colloidal chitin also confirmed that these two C-terminal fusion chimeras were more active than the other chimeras. The chimeras ChiD+PKD and PDC displayed improved TG, in terms of increased quantity of TG products produced and also with the extended duration of TG. It could be either the orientation of auxiliary domains or the final fold of chimeric proteins influencing the hydrolysis/TG activities, being confirmed through structural analysis. The *Sp*ChiD fusion chimeras with improved hydrolytic activity would be useful for efficient recycling of chitin bio-mass.

## Supporting Information

S1 Table Details of primers and templates used for generation of *Sp*ChiD fusion chimeras. (DOC)
