# Supplementary material for: Catalytic Efficiency of Chitinase-D on Insoluble Chitinous Substrates Was Improved by Fusing Auxiliary Domains
Source: PLoS One. 2015 Jan 23;10(1):e0116823. doi: 10.1371/journal.pone.0116823 (PMC4304778; doi:10.1371/journal.pone.0116823)
Supplement: S1 Table — (DOC) [file pone.0116823.s001.doc]

| **S.No** | **Name of the primer** | **Primer sequence (5’-3’)** | **Template used for PCR** | **Name of the gene amplified** | **Amplicon size (kb)** | **Primer combination for fusion** | **Name of the plasmid** |
| --- | --- | --- | --- | --- | --- | --- | --- |
| 1a. | CBP21 Fp | AAT AAC CAT GGT TCA CGG CTA TGT CGA AAC | pCBP21-pET22b(+) | CBP21 with overhang of ChiD | 0.51 | CBP21 Fp  &  Chi D Rp  (~1.7kb) | CBP+ChiD  pET22b(+) |
|  | CBP21RpO | agc cat gcc ggc acc TTT AGT CAA ATT AAC |  |  |  |  |  |
| 1b. | Chi D IFp | GGT GCC GGC ATG GCT CAT GCC GCT TCT TAC | pChiD-pET22b(+) | ChiD without RS at the N-terminus | 1.2 |  |  |
|  | Chi D Rp | AAT AAC TCG AGC TGT TTC CCG CCG TTA ATC C |  |  |  |  |  |
| 2a. | Chi D Fp | TAA TAC CAT GGG TGC CGG CAT GGC TCA TG | pChiD- pET22b(+) | ChiD with overhang of CBP21 | 1.2 | Chi D Fp  &  CBP21 Rp  (~1.7kb) | ChiD+CBP  pET22b(+) |
|  | Chi D RpO | TTC GAC ATA GCC GTG CTG TTT CCC GCC GTT |  |  |  |  |  |
| 2b. | CBP21 IFp | CAC GGC TAT GTC GAA ACC CCG GCC AGT CGT | pCBP21- pET22b(+) | CBP21 without RS  at the N-terminus | 0.51 |  |  |
|  | CBP21 Rp | GCA TAC TCG AGT TTA GTC AAA TTA ACG TC |  |  |  |  |  |
| 3a. | PKD Fp | CAA TAA CCA TGG CCG TAC CGG GTA AGC CTA C | pChiA- pET22b(+) | PKD with overhang of ChiD | 0.4 | PKD Fp  &  Chi D Rp  (~1.6kb) | PKD+ChiD  pET22b(+) |
|  | PKD RpO | agc cat gcc ggc acc gcc gga atc ctg ctt |  |  |  |  |  |
| 3b. | Chi D IFp | GGT GCC GGC ATG GCT CAT GCC GCT TCT TAC | pChiD- pET22b(+) | ChiD without RS at the N-terminus | 1.2 |  |  |
|  | Chi D Rp | AAT AAC TCG AGC TGT TTC CCG CCG TTA ATC C |  |  |  |  |  |
| 4a. | Chi D Fp | TAA TAC CAT GGG TGC CGG CAT GGC TCA TG | pChiD- pET22b(+) | ChiD with overhang of PKD | 1.2 | Chi D Fp  &  PKD Rp  (~1.6kb) | ChiD+ PKD pET22b(+) |
|  | Chi DARpO | CTT ACC CGG TAC GGC CTG TTT CCC GCC GTT |  |  |  |  |  |
| 4b. | PKD IFp | GCC GTA CCG GGT AAG CCT ACG CTG GCC TGG | pChiA- pET22b(+) | PKD without RS at the N-terminus | 0.4 |  |  |
|  | PKD Rp | GCA TAC TCG AGG CCG GAA TCC TGC TTA TA |  |  |  |  |  |
| 5a. | PKD Fp | CAA TAA CCA TGG CCG TAC CGG GTA AGC CTA C | PKD+ChiD  pET22b(+) | PKD+ChiD with overhang of CBP21 | 1.6 | PKD Fp  &  CBP21 Rp  (~2.1kb) | PKD+ChiD+CBP  pET22b(+) |
|  | Chi D RpO | TTC GAC ATA GCC GTG CTG TTT CCC GCC GTT |  |  |  |  |  |
| 5b. | CBP21 IFp | CAC GGC TAT GTC GAA ACC CCG GCC AGT CGT | pCBP21- pET22b(+) | CBP21 without RS  at the N-terminus | 0.51 |  |  |
|  | CBP21 Rp | GCA TAC TCG AGT TTA GTC AAA TTA ACG TC |  |  |  |  |  |
| 6a. | CBP21 Fp | AAT AAC CAT GGT TCA CGG CTA TGT CGA AAC | CBP+ChiD  pET22b(+) | CBP21+ChiD with overhang of PKD | 1.7 | CBP21 Fp  &  PKD Rp  (~2.1kb) | CBP+ChiD+PKD  pET22b(+) |
|  | Chi DARpO | CTT ACC CGG TAC GGC CTG TTT CCC GCC GTT |  |  |  |  |  |
| 6b. | PKD IFp | GCC GTA CCG GGT AAG CCT ACG CTG GCC TGG | pChiA- pET22b(+) | PKD without RS at the N-terminus | 0.4 |  |  |
|  | PKD Rp | GCA TAC TCG AGG CCG GAA TCC TGC TTA TA |  |  |  |  |  |

Table S1 - Details of primers and templates used for generation of *Sp*ChiD fusion chimeras
